# Endogenous and Exogenous Stem/Progenitor Cells in the Lung and Their Role in the Pathogenesis and Treatment of Pediatric Lung Disease

**DOI:** 10.3389/fped.2016.00036

**Published:** 2016-04-14

**Authors:** Sandra Leibel, Martin Post

**Affiliations:** ^1^Program of Physiology & Experimental Medicine, The Hospital for Sick Children, Toronto, ON, Canada; ^2^Department of Physiology, University of Toronto, Toronto, ON, Canada; ^3^Department of Pediatrics, University of California San Diego, San Diego, CA, USA

**Keywords:** stem cells, lung, developmental biology, therapeutics, lung diseases

## Abstract

The lung is a complex organ with a vast surface area whose main function is to release cellular waste to be exhaled and to replenish the supply of oxygen to the tissues of the body. The conduction of air from the external environment is not without risks, and the lung contains many specialized epithelial cell subtypes that are protecting the lung from foreign material and injury. Specialized cell subtypes are produced during lung development in the fetus as well as postnatally and injury to them due to genetic disease, premature birth, or postnatal environmental injury may lead to devastating disease. Chronic diseases, such as bronchopulmonary dysplasia, cystic fibrosis, and pulmonary arterial hypertension, contribute significantly to morbidity and mortality worldwide, yet successful interventions are often limited. Stem/progenitor cells have emerged as a potentially new preventative or therapeutic option. They are generally defined by the ability to undergo self-renewal and give rise to more differentiated cells. They are important in the early development of embryonic structures and organ differentiation *in utero*. Postnatally, they function in continued growth, maintenance, and regeneration. Clinically, the immunomodulatory properties of some classes of stem/progenitor cells avoid the major obstacle of immunological rejection seen in organ transplantation and other cell therapies. This review highlights some known human progenitor/stem cells and the most recent advances in stem cell therapies both *in vivo* and *in vitro* to prevent and treat pediatric lung disease.

## Lung Development

**Figure 1:**
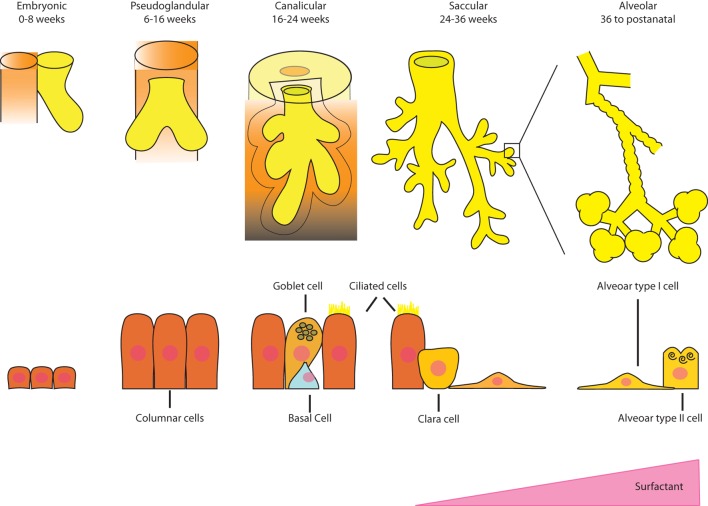
**Schematic diagram of the complexity of the lung structure during human lung development**.

The lung develops through five stages in the human (Figure 1), and a multitude of genes and transcription factors are involved in the mediation of each phase. In the embryonic phase, endoderm, specifically anterior endoderm, gives rise to the lung, which begins with the formation of a groove in the ventral lower pharynx, which then buds to form the true lung primordium. (1). The interaction between the epithelium and mesenchyme is critical in this early stage (2). Septation of the tracheoesophageal tube separates the respiratory tree from the gastrointestinal tract. Transcription factors are important in this process and are activated early in lung progenitor cells (3).

In the pseudoglandular stage, branching morphogenesis of the epithelium into the surrounding mesenchyme dominates and forms the entire air-conducting bronchial tree up to the terminal bronchioli. During this period, the developing epithelium begins to secrete fluid into the budding airways, which is important for the further growth of the primordial lung (4). Branching occurs within a preprogrammed set of rules and is governed by the balance of attraction and inhibition of FGF10 from the mesenchyme along with multiple other regulators (2). Embryonic stem/progenitor cell functions have been uncovered in the pseudoglandular stage, such as a multipotent progenitor population expressing the inhibitor of differentiation 2 (Id2) in the mouse (5). This transcriptional regulator is very strongly expressed in distal epithelial tips of the branching sites that are thought to give rise to both proximal conducting airway and distal alveolar epithelial cell types. Another lung progenitor cell, this time from the mesoderm, namely, cardiopulmonary mesoderm progenitor (CPP) cells, arises from the cardiopulmonary mesenchyme and gives rise to cardiomyocytes, endocardium, pulmonary vascular, and airway smooth muscle cells (6). Interestingly, CPP cells do not rely on parallel epithelial lung development for their growth as previously thought.

The canalicular stage comprises the branching of the respiratory portion of the lung from the terminal bronchioli. These air spaces form an acinus comprising respiratory bronchioles and the alveolar ducts. In this stage, the capillaries invade the mesenchyme and begin to surround the acini. Type I and II pneumocytes appear, proliferate, and surfactant begins to be secreted. It is late in this stage when a preterm newborn has the capacity to survive with assisted ventilation in the extra uterine environment.

At approximately 25 weeks, whole clusters of sacs extend from the terminal bronchiole, defining the saccular stage. These saccules are coated with type I and II pneumocytes, and they are divided by primary septa, which are thick and contain two layers of capillaries. The interstitial space or matrix becomes rich with a variety of cell types as well as collagen and elastic fibers. Bipotential alveolar progenitors in the mouse develop into ATII and alveolar type I (ATI) cells but the signals and timing are still unknown as whether there is an equivalent progenitor in the human (7).

The final stage beginning in the late third trimester is defined by the alveolarization of the lung. Secondary septa begin to form, and the basement membrane of the capillary endothelium and the saccular epithelium merge to form a thin barrier. A large number of small protrusions form along the primary septa, becoming larger and subdivide the sacculi into smaller subunits, the alveoli, which are delimited by secondary septa (8). This phenomenon continues well into extra uterine life.

Vascular development of the lung consists of vasculogenesis (*de novo* formation of the vascular plexus from mesodermal progenitor cells) and angiogenesis (sprouting of endothelial cells from pre-existing vessels to form new tubes) (9). Endothelial cells of the newly formed tubes recruit pericytes, which wrap around endothelial tubes and induce stabilization and maturation (10). Tight regulation of this interaction is required for normal vascular development, and disruption of this process may lead postnatal lung disease.

Finally, an integral part of the developing lung is the extracellular matrix (ECM). This is a complex network of components that have a structural, biochemical, and mechanical function. In lung development, the ECM serves as a scaffold that directs cell migration and differentiation, becoming more complex with development (11). Any alterations in the structure of the ECM whether through premature developmental arrest or injury will greatly alter the function of the lung.

This complicated process of lung development has been well studied in animal models, and many of the genetic and molecular factors of the major stages, as well as some stem cell populations have been defined with respect to human lung development, although much is still unknown (12).

## Lung Disease Resulting from Disrupted Lung Development

Bronchopulmonary dysplasia is the sequela of preterm birth. The hallmark of this disease is alveolar growth arrest and abnormal lung vascular growth (13). This alveolar growth arrest is both a product of the disruption of secondary septation formation as well as a matrix that has not been fully formed. The formation of the pulmonary capillaries destined to appose the alveoli is also stunted. The etiology is multifactorial including infection, hyperoxia, and volutrauma superimposed on an immature lung with decreased defenses (14). At a cellular level, these abnormalities are presumed to be due to defective elastogenesis and ECM remodeling (15), altered alveolar epithelial–mesenchymal interactions, and impaired development of lung microvasculature (16). These changes may have an effect on the intrinsic stem/progenitor cell populations, making it difficult to repair the fragile young lung.

Idiopathic pulmonary arterial hypertension (IPAH) is a rare disorder of unknown etiology clinically defined by raised pulmonary artery pressures involving pathological changes in precapillary pulmonary artery. Although there has been significant progress to improve the morbidity and mortality of this disease, it remains a serious condition, which is extremely challenging to manage (17).

These diseases have been a focus of human stem cell treatment and will be highlighted below.

## Stem/Progenitor Cells in Homeostasis/Injury Repair

**Figure 2:**
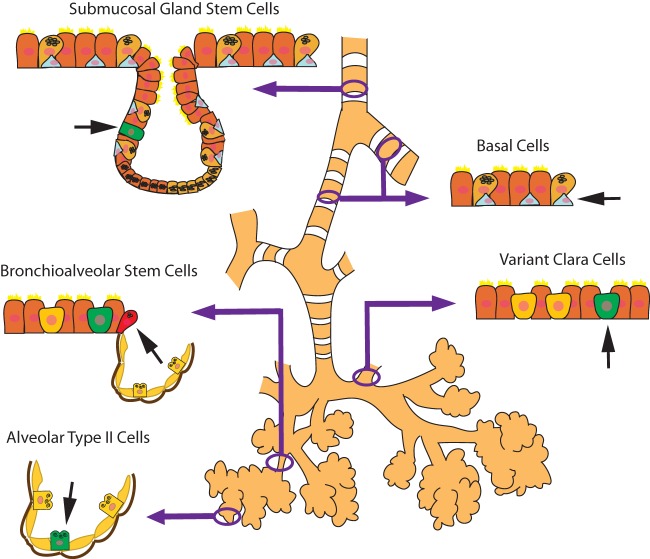
**Schematic diagram of the microenvironmental niches that may contain lung stem/progenitor cells**.

Although stem cells/progenitors are important in the development of the lung, they also play an important role in lung regeneration and repair. Human tissue regeneration *via* native or recruited stem cell populations involves several mechanisms, which are regionally distinct and dependent on the type of injury (18). Constantly renewing organs, such as the hematopoietic system, have stem cells that are unspecialized, have a low rate of division, and are located in specialized “niches” (19). The lung is an organ that is slow to regenerate but can initiate rapid repair after injury. It is postulated that there are niches in the lung that house quiescent progenitor cells that have the potential to self-renew and generate progeny to regenerate the lung epithelium specific to that location (Figure 2) (20). In the proximal airway, located in the gland duct and on the surface in the intercartilaginous zone, are basal cells, which are multipotent stem cells in the tracheobronchial region that can both self-renew and give rise to ciliated and secretory lineages during postnatal growth, homeostasis, and repair following damage to the epithelium (21). Non-ciliated secretory Clara cells (or Club cells) and variant Clara cells, which are located at the bronchiolar–alveolar duct junction and associated with the neuroepithelial body, give rise to ciliated and secretory cells (22, 23). A small number of cells in the bronchioalveolar duct junction (BADJ) co-express SCGB1A1 (a Clara cell marker) and Surfactant protein C (SFTPC), a protein that is expressed by ATII cells in the alveoli. It has been proposed that these “dual positive” cells are bronchioalveolar stem cells or BASCs (24), which apparently give rise to bronchiolar and alveolar cell types in culture. In the peribronchial region, basal cells appear during repair and proliferate in the distal lung, giving rise to functional alveoli (25).

Finally, ATII cells in the alveoli give rise to ATI cells although the rate of conversion depends on whether the cells are in steady state (slow rate) or during repair after injury (fast rate) (18, 26). Although more is known of the stem cell niches and cell populations in the mouse lung, there have been some advances in the human lung as well. Investigators have discovered c-Kit^+^ cells in human lungs that are undifferentiated, self-renewing, clonigenic, and multipotent *in vitro*. C-kit is a transmembrane tyrosine kinase receptor for stem cell factor (SCF) and has a diverse range of biological functions including cell proliferation, differentiation, migration, and survival (27). Injection of the c-kit^+^ cells into a mouse model of focal lung injury regenerates lung components as diverse as bronchioles, alveoli, smooth muscle, and pulmonary vessels (28). This is the first evidence of a “lung stem cell” that can regenerate both endodermal and mesenchymal cell lineages, although these findings have been disputed (29). It has been challenging to define the properties of lung stem/progenitor cells due to the lungs complexity and diversity of cell types as well as the slow turnover of the respiratory epithelium. The regenerative capacity of many of these cells is also not solely determined by their intrinsic potential. The microenvironment of their specific niche, including the ECM, accessory cells, and many signaling factors, are also important regulators (30, 31) and warrant further investigation.

Various stem/progenitor cells that function in lung repair reside in other areas of the body and are recruited in times of injury and inflammation. Cells from the bone marrow, blood, adipose tissue, placenta, and umbilical cord have been shown to structurally engraft in the airway (32, 33) as well as the pulmonary vasculature. Another potential mechanism is that bone marrow-derived cells are recruited to the lung upon injury and exert their regenerative effects *via* a paracrine function (34, 35). One population is the mesenchymal stromal cells (MSCs). These cells are multipotent and have a diverse but restricted ability to differentiate to a specific lineage. They appear to function in a paracrine manner with minimal engraftment, interact with the innate and adaptive immune systems (36, 37), and aid in lung repair and regeneration *via* secretion of cytokines and growth factors to restore alveolar epithelial and endothelial permeability (38–41). Chang et al. (42) used a hyperoxia rat model of BPD to determine the best administration route of human cord blood-derived MSCs. They exposed rat pups to 95% oxygen from birth and at day 5 delivered the MSCs either intratracheally or intraperitoneally. They showed that the intratracheally transplanted MSCs were better in preventing alveolar growth arrest and alleviating fibrotic changes in the lungs of oxygen challenged rat pups than the intraperitoneally administered cells. These finding have been corroborated by Zhang et al. (43) using a similar hyperoxic BPD model. They treated the rat pups with bone marrow-derived MSCs 7 days after the hyperoxic insult and saw a decrease in alveolar apoptosis. They concluded that the MSC’s protective function was due to stimulation of mediators that participated in tissue repair. Thebaud’s group (34), using the same BPD rat model, showed that human umbilical cord-derived MSCs partially prevented and rescued lung function and structure, although cell engraftment was low. Postulating a paracrine effect of MSCs, they derived MSC conditioned media, which after infusion into the hyperoxia exposed animal, showed similar therapeutic benefits as the cells themselves. They also looked at the lungs of these rats 6 months after the intervention and showed a persistent improvement in lung capacity and lung structure. Kourembanas’ group evaluated the pulmonary abnormalities in BPD. They found that a single dose of MSC conditioned media in hyperoxia-exposed newborn mice reversed the hyperoxia-induced parenchymal fibrosis, partially reversed alveolar injury, normalized lung function defined as airway resistance and dynamic lung compliance, fully reversed the moderate right ventricular hypertrophy, and attenuated peripheral pulmonary artery muscularization associated with hyperoxia-induced BPD (44). There are many investigators studying the biology of MSCs on lung injury, and for a full review of animal models of BPD and therapeutic use of MSCs please, see the review by O’Reilly et al. (45).

Endothelial progenitor cells (EPCs) come in two flavors: “early” EPCs, which have hematopoietic surface markers, secrete pro-angiogenic factors, and have limited differentiation ability; “late” EPCs [or endothelial colony-forming cells (ECFCs)] that have no hematopoietic surface markers, do not secrete pro-angiogenic factors, make endothelioid tubes *in vitro*, grow late in culture, and are important in replacing damaged endothelium (46). These cells exert their therapeutic effects *via* direct differentiation and engraftment into the vasculature of the lung and secretion of factors that mobilize endothelial and progenitor cells. Kung et al. (47) seeded adult peripheral blood ECFCs onto acellular human skin and transplanted the celluarized skin scaffolds into immunocompromised mice. They formed functional human endothelial cell vessels, which had anastomosed with the circulation of the mouse. Shepherd et al. (48) compared umbilical cord blood-derived ECFCs, adult peripheral blood-derived ECFCs, or human umbilical vein endothelial cells (HUVECs) in a similar model. The umbilical cord blood-derived ECFCs exhibited a greater human vessel density than the other ECFCs. These studies show that ECFCs represent a promising source for vascular regeneration.

Amniotic fluid stem cells (AFSCs) have been used since the late 90s in animal models to study their function in a variety of organ systems. Human AFSCs are fetal-associated cells that are multipotent and can differentiate into all germ layers and can be easily and ethically obtained from amniocentesis specimens. Intratracheal injection of human AFSCs into a rabbit model of congenital diaphragmatic hernia showed improved lung density and function (49). Carraro et al. (33) injected human AFSCs locally to the murine distal lung and saw integration into the epithelium and expression of the early lung marker NKX2-1. After oxidative injury, the injected cells expressed both NKX2-1 and SFPTC (ATII cell marker). These same investigators deduced that the hAFSCs attached within the ATII wound and expedited repair through the secretion of cytokines. The damaged milieu also allowed healing through the differentiation of these cells into distal alveolar epithelium (50). Although no clinical trials using hAFSCs in lung disease have been done, they show promising future use.

Human amnion epithelial cells (hAECs) are found in the lining of the placenta and are able to develop into all the germ layers, possess regenerative and anti-inflammatory properties, and display low immunogenicity. Many animal models of lung injury have been used to study the effects of these cells. Hodges et al. (51) looked at three groups of lambs, and evaluated their lung injury after being ventilated *in utero* alone or with intravenous and intratracheal administration of hAECs at 110 days of gestation. The lambs were replaced into the womb, and after a week, removed and evaluated. The investigators found that the stem cells mitigated ventilation-induced lung injury, engrafted onto the lung and differentiated into ATI and ATII cells. Vosodoganes et al. (52) showed in an intrauterine LPS-induced model of lung inflammation in fetal sheep that hAECs significantly attenuated the fetal pulmonary inflammatory response after being administered intravenously, but that they did not improve lung structure. Other investigators studying the anti-inflammatory effect of hAECs on bleomycin-treated mice confirmed that hAEC’s attenuated the inflammatory response and improved lung function (53, 54). Murphy et al. (55) found that hAECs formed three-dimensional structures, expressed the CFTR gene and protein after culture in Small Airway Growth Medium (SAGM) and possessed functional iodide/chloride [I(−)/Cl(−)] ion channels. This showed that hAECs may be a new source for the development of a cellular therapy for cystic fibrosis.

These cells have not yet been used in clinical trials due to the lack of large scale production of clearly defined amnion epithelial cells.

## Clinical Application of Stem/Progenitor Cells in Pediatric Lung Disease

Animal models have been important in elucidating many of the potential repair mechanisms of a variety of stem/progenitor cells but, even without knowing all underlying mechanisms or risks, their clinical application for use in BPD and other pediatric lung diseases has exploded.

Mesenchymal stromal cells can be obtained from multiple tissues of the body in adults as well as children and large supplies are known to come from the products of pregnancy including cord blood, placenta, and amnion. Cord blood MSCs are generating a lot of interest since they can be obtained without ethical constrictions, can be easily harvested, and are superior in their healing capabilities than adult bone marrow cells (56). Their widespread use in clinical trials has also been due to their immunomodulatory behavior. MSCs express low levels of major histocompatibility complex (MHC) class I molecules and no MHC class II molecules, allowing them to be poorly immunogenic. They also do not express costimulatory molecules involved in the activation of T cell for transplant rejection (57). All these characteristics have been delineated in both adult and fetal MSCs and make them strong candidates for cellular therapies. Clinically, MSCs have been used successfully in many disease processes, but in the lung, a phase II trial for moderate to severe COPD (58) showed no therapeutic effect, although it was not powered for clinical efficacy. In patients that received the MSCs, there were no changes from baseline except a decrease in CRP, a marker of inflammation, and there were no harmful side effects that differed between the two groups. In preterm infants with BPD, a phase I trial in Korea evaluated the safety and the efficacy of human umbilical cord blood-derived-mesenchymal stromal cell (hUCB) treatment in premature infants with BPD. Intratracheal MSC transplantation was performed in nine preterm infants, with a mean gestational age of 25 weeks. BPD severity was lower in the transplant recipients, and rates of other adverse outcomes did not differ between the comparison group and transplant recipients. They concluded that intratracheal transplantation of allogeneic hUCB-derived MSCs in preterm infants was safe and feasible, and warranted a larger and controlled phase II study (59). Other pulmonary diseases in which these cells are currently being investigated clinically are asthma, idiopathic pulmonary fibrosis (IPF), and bronchiolitis obliterans syndrome (BOS) (60). The barriers of clinically using MSCs include not knowing the safest and efficacious route, optimal dose, and the incomplete understanding of mechanism of action. These cells are also heterogeneous, and a well-defined clinically validated product is not available.

Angiogenesis is crucial for normal postnatal alveolar development (61). EPCs and ECFCs are circulating peripheral cells that travel to ischemic sites and augment angiogenesis *via* paracrine effects (62, 63). Experimentally, a depletion of EPCs in the blood, bone marrow, and lungs of neonatal mice was detected after exposing mice to hyperoxia, but there was a twofold increase of EPCs in the lungs of adult mice exposed to hyperoxia, suggesting the migration of these cells to the lung for repair (64). This was not consistent in human babies (65). Thirty-six preterm neonates at risk of lung injury were evaluated for serum levels of EPCs, and it was found that levels of EPCs did not affect the risk of developing BPD. Follow-up blood draws at 36 weeks postmenstrual weeks showed that the levels of the EPCs were preserved after delivery. Another group of investigators studied ECFCs in the cord blood of 98 preterm babies for the proportion of circulating cells at birth and up to a week after, using flow cytometry (63). They found that ECFCs in cord blood were lower in infants who later developed BPD. This was felt to contribute to the vascular immaturity seen in this disease.

Human trials have been performed to examine whether exogenously transplanted EPCs to patients with vascular lung disease are beneficial. Zhu et al. (66) used autologous EPC transplantation in children with IPAH. Thirteen children received IV autologous EPCs and after 12 weeks, showed improvements in exercise capacity and pulmonary hemodynamics. Although this was just a pilot study, the clinical use of EPCs in childhood IPAH seems safe and feasible. A Canadian phase I study using EPCs transfected with endothelial nitric oxide synthase in seven patients has recently been completed (NCT00469027), and the final results are pending. Clinical use is hampered by the lack of large-scale production of clearly defined EPCs and ECFCs, and their method of action is even less understood than MSCs.

Table 1 summarizes the clinical applications of the various cell types in pediatric lung disease.

**Table 1:** **Pediatric clinical trials using a variety of stem/progenitor cells for the treatment of pulmonary diseases**.

Disease	Stem cell	Phase	Route	Outcomes	Author/trial
BPD	hUCB-derived MSCs	I	Intratracheal	Lower BPD severity	Chang et al. (59)
BPD	hUCB-derived MSCs	II	Intratracheal	Recruiting	NCT01897987
BPD	hUCB-derived MSCs	I/II	Intratracheal	Recruiting	NCT02381366
Bronchiolitis obliterans	Mesenchymal stromal cells	I	IV	Active	NCT01175655
IPAH	Autologous endothelial progenitor cells	I	IV	Significant improvements in exercise capacity, NYHA functional class, and pulmonary hemodynamics	Zhu et al. (66)

*hUCB-derived MSCs, human umbilical cord blood-derived mesenchymal stem cells*.

## *In Vitro* Stem Cell-Derived Lung Cells

Human embryonic stem cells (hESC) and induced pluripotent stem cells (hiPSCs) are pluripotent cells that can be differentiated into any tissue in the body. They are naturally derived from human embryos (hESC) (67) or derived from differentiated tissues such as skin or blood after transfection with a specific set of transcription factors (hiPSCs) (68). Both cell types are easy to maintain in culture and can be produced in large quantities for clinical application. By recapitulating lung development in culture, these cells can be coaxed into differentiating into the vast array of epithelia subtypes although lung generation from stem cells has lagged behind other tissue types (12). There are many protocols for differentiating stem cells into lung cells, but the induction of definitive endoderm from the stem cell is mostly achieved with a high concentration of activin A, a known signaling molecule in early lung development. Anterior foregut endoderm is then derived using a combination of small molecules and cytokines (69). These cells are then exposed to a variety of cytokines in order to reach a lung progenitor phenotype, expressing the transcription factor NKX2-1, the first transcription factor signaling the appearance of lung progenitor cells (70). Longmire et al. (71) used Nkx2-1–GFP reporter mouse ESCs to sort out the Nkx2-1-positive cells, and after treating them in culture, there was expression of both proximal and distal lung cell markers. Multiple researchers have then exposed human lung progenitor cells to a cocktail of exogenous signals in a variety of culture conditions and were able to show markers expressing basal, ciliated, and mucus cells (72), distal ATI and ATII (70), mature ciliated epithelium using airway liquid interface (ALI) culture (73), and three-dimensional spheroids expressing multiple lung subtype markers, both proximal and distal (74). Ghaedi et al. (75) went a step further and seeded SPC^+^ ATII cells derived from hiPSCs onto a decelluralized lung matrix onto which they adhered and proliferated. The Otts lab recently reported the regeneration of functional pulmonary vasculature by repopulating the vascular compartment of decellularized rat and human lung scaffolds with human cells (76).

The clinical application of these stem cell-derived lung cells into injured lung tissue has many possibilities, including correcting the genetic mutations in patient specific cells, and eventually replenishing the injured lung with the corrected cells, without immunologic rejection, although this still has a long way to go. Safety, mode of delivery, efficiency, large scale production, and purity have to first be evaluated in animals successfully. One strategy that has been evaluated in the mouse and human respiratory system is decellularization using the intact acellular matrix of the lung as a base for fresh lung progenitors. Recently, Shojaie et al. (77) evaluated the role of the lung ECM in differentiating stem cell-derived definitive endoderm into mature airway epithelia. Clinically, a Swedish group decellularized an adult human donor trachea, which was then colonized by epithelial cells and mesenchymal stem cell-derived chondrocytes. This graft was then used to replace the recipient’s left main bronchus. At the 5-year follow-up, the tissue-engineered trachea was patent, well vascularized, completely recellularized with respiratory epithelium, and had normal ciliary function and mucus clearance. No stem cell-related teratoma formed, and no anti-donor antibodies developed (78). Another group, led by Di Coppi, treated a pediatric patient with congenital tracheal stenosis with a decellularised cadaveric donor tracheal scaffold and seeded it with bone marrow mesenchymal stem cells. The graft revascularized within 1 week after surgery, the patient had a normal chest CT scan and ventilation–perfusion scan after 18 months after surgery, and at 2-year follow-up, he had a functional airway (79).

Once the derivation of mature and functional lung epithelial cells that mirror their *in vivo* counterparts is possible and the best route and both short- and long-term safety are guaranteed, these patient-specific derived lung cells can potentially be used clinically to populate severely injured lung tissue in various pediatric diseases, without the immunologic and ethical burden of lung transplantation.

## Conclusion

In conclusion, although the field of stem cell lung biology is expanding rapidly and animal models of various pediatric lung diseases are providing insight to other molecular mechanisms of lung injury, these diseases remain a chronic burden on the child despite symptomatic therapy. Evidence has shown that damage to endogenous stem cells may be contributing to the risk and etiology of the disease, and exogenously administered stem cells may offer new possibilities in preventing or curing these diseases. Continued work in stem cell biology, lung development, and the underlying disruption of normal development will add to our knowledge, and when applied clinically, will provide us with successful protocols to finally prevent or treat these diseases.

## Author Contributions

Conception and design, writing, review, and/or revision of the manuscript: SL and MP. Study supervision: MP.

## Conflict of Interest Statement

The authors declare that the research was conducted in the absence of any commercial or financial relationships that could be construed as a potential conflict of interest.
